# Ultrasound systems integrated into industrial continuous-flow extraction process: Cultivar-based assessment for improved yield and quality

**DOI:** 10.1016/j.ultsonch.2025.107451

**Published:** 2025-07-03

**Authors:** Antonia Tamborrino, Gianluca Veneziani, Alessandro Leone, Antonio Berardi, Sonia Esposto, Maurizio Servili

**Affiliations:** a Department of Agricultural and Environmental Science, University of Bari Aldo Moro, Via Amendola 165/A, 70126 Bari, Italy; b Department of the Science of Agriculture, Food and Environment, University of Perugia via S. Costanzo, 06126 Perugia, Italy

**Keywords:** High power ultrasound, Malaxation, Viscosity, Olive oil quality, Extractability, Rheology

## Abstract

In the agri-food sector, the olive oil mill boasts a consolidated process technology, supported by to in-depth research studies, for the extraction of olive oil. The market and consumers now demand continuous optimization of production processes, greater extractability of the product and superior quality of Extra Virgin Olive Oil (EVOO).

Emerging technologies, based on physical treatment processes applicated to olive pastes, such as pulsed electric fields (PEF), microwave-assisted extraction and ultrasound-assisted extraction (UAE), integrated into conventional extraction processes, have demonstrated increased extractability, reduced production costs and time, and improved final product quality.

Starting from the results of previous studies, this implemented an experimental test plan with a US plant installed before the malaxer phase, processing olive paste, performing tests on different olive cultivars: Coratina and Nocellara del Belice typical of two different areas of Italy: Puglia and Sicily.

The results demonstrated a 3% increase in extractability and an improvement in the qualitative values of the EVOO phenolic composition.

## 1 Introduction

In recent years, there has been a surge in interest in natural extracts derived from plants and the production of high-quality agri-food products rich in bioactive compounds. This trend spans across agronomy, pharmaceuticals, and manufacturing, largely driven by consumers’ growing awareness and expectations regarding food quality and health benefits. Unlike in the past, when the focus was on maximizing production volumes, producers are now prioritizing technological advancements to enhance product quality and meet consumer demands.

The olive oil industry stands out as a strategic sector in the Italian national economy, representing one of the largest agri-food industries. This sector produces various categories of olive oil using physical or mechanical extraction methods, allowing for oils that can be consumed without additional processing[1,2]. Each category of olive oil corresponds to different quality levels, the most prized and commercially valuable category is the Extra Virgin Olive Oil (EVOO) due to its high-energy food, delivering approximately 899 kcal per 100 ml and comprising 99.9 % lipids, particularly monounsaturated fatty acids. Additionally, its’s richness in polyphenols, especially oleuropein and hydroxytyrosol, two of the most potent natural antioxidants. On one hand these compounds contribute to the prevention and management of several diseases, including neurodegenerative and cardiovascular conditions [3]. On the other hand, from an organoleptic perspective, phenolic compounds impart the bitter and spicy flavors to Extra Virgin Olive Oil (EVOO), shaping its unique sensory profile alongside volatile compounds[4].

Extra Virgin Olive Oil (EVOO) production involves solely mechanical processes, beginning with crushing the olives to rupture cell walls and release oil droplets from the mesocarp[5,6]. The next crucial step, malaxation, involves low-speed kneading (20–30 rpm) to encourage the coalescence of small oil droplets into larger ones, easing their separation through centrifugation. Malaxation affects both the yield and sensory qualities of the oil, necessitating careful control over time and temperature to achieve high oil quality and extraction efficiency[7,8]. The significant residue in the olive oil extraction process is olive pomace, which consists of skin, pulp, and fragments of stone and seed, amounting to about 80 % of the original fruit weight [9]. This pomace retains most of the phenolic compounds, with over 95 % of these bio-compounds remaining in the pomace rather than the oil itself. When improperly disposed of these compounds can have negative environmental impacts [10]; Čepo, et al., 2018).

In light of this, consumer interest in EVOO with distinct fruity profiles and his ability to distinguish an oil organoleptically defining a vegetal and fresh aroma as synonymous for excellent oil quality, has driven research into innovative techniques to improve oil yield and quality, as well as environmental sustainability.

Continued research and innovation are crucial for maintaining technological leadership and meeting evolving consumer and environmental standards.

Emerging technologies have been used in the (EVOO) to improve extraction process such as, pulsed electric fields (PEF)[11,12,13,8,14,15,16]and microwave-assisted extraction [17,18,19,[20], [21], [22]], ultrasound-assisted extraction (UAE)[17,23,24,6,18,25,19,[20], [21], [22]]. These technologies have shown promise results in enhancing EVOO production. They have been implemented in conventional extraction processes to increase extraction efficiency, reduce processing time, lower production costs, and improve the final product’s quality including extended shelf life. However, most conventional methods rely on extending malaxation time or increasing temperature to boost oil yield [26,27,28,29,30]; at the same time, such practices can degrade oil quality and reduce antioxidant stability due to enzyme activity in the olive paste, impacting free fatty acid content, peroxide value, and phenol levels[31,32,33,30].

Among these unconventional techniques, ultrasound (UAE) has emerged as a particularly effective method for improving both the quality and yield of EVOO. It offers a cost-effective and efficient alternative to traditional extraction methods, which can lead to the loss of phenolic compounds through oxidation and other reactions[34].

Ultrasound (UAE) technologies can be classified as high-power ultrasound (HPU, 20 kHz − 120 kHz or more) and diagnostic ultrasound (above 1 MHz)[[20], [21], [22]], it utilizes sound waves (20 Hz to 20 kHz) to create thermal and mechanical effects on olive paste. The thermal effect occurs when ultrasound waves convert kinetic energy to thermal energy, while the mechanical effect arises from cavitation a process of bubble formation, growth, and implosion under high negative pressure. Cavitation disrupts cell walls, facilitating the release of soluble compounds and improving mass transfer within olive tissues enhancing extraction efficiency.

The aim of this study is to evaluate the application of ultrasound technology in olive oil extraction, focusing on its impact on process efficiency, oil yield, and quality parameters. The research seeks to investigate the role of ultrasonic treatment in enhancing the release of oil from olive paste by promoting cell wall disruption and facilitating the coalescence of oil droplets. Additionally, the study will assess the potential benefits of ultrasound in reducing processing times, improving thermal efficiency, and preserving the sensory and nutritional properties of the extracted olive oil.

In addition to ultrasound, more recent approaches have combined multiple technologies to further improve oil extraction. For instance, the integration of a mixing-coil heat exchanger with both microwave and ultrasound technologies has been shown to significantly improve the efficiency of oil extraction process while preserving the oil's phenolic profile. This combined method enhances the temperature control during extraction, which aids in the rupture of olive cells and maximizes oil yield without compromising quality. Studies by Leone et al. [17,18] have demonstrated that this innovative combination of ultrasound and microwave technologies offers a promising alternative for EVOO production, improving both yield and oil quality while minimizing oxidative degradation of phenolic compounds. Additionally, Amirante and Paduano [23] highlighted the effectiveness of ultrasound in enhancing extraction efficiency, which further supports the move toward more sustainable, cost-effective olive oil production methods.

## 2 Materials and methods

### 2.1 Industrial olive oil extraction plant and raw material

The experimental tests were carried out in two commercial olive oil mills: Agricola Malerba, Terlizzi (BA), Puglia, Italy and Frantoio Cutrera, Piano Dell’Acqua (RG), Sicily, Italy.

The Agricola Malerba mill, Fig. 1a, was composed of a defoliator, a washing machine, a hammer crusher (model Frangolea, Barracane s.r.l., Modugno, BA, Italy), a group of two malaxer machines (model Gramola 1400, Barracane s.r.l., Modugno, BA, Italy) arranged in series with a cavity pump for discharging the malaxer and feeding a decanter model Megala 650 CI (Barracane s.r.l., Modugno, BA, Italy) and a liquid/liquid vertical plate centrifuges (model Grande, Barracane s.r.l., Modugno, BA, Italy). The decanter during the experimental tests was set at 2.5-phases without water added producing a pomace rich in rigid solids from pit shells with lower humidity (similar to that from the conventional three-phase decanter) and a wet pulp known as “patè” containing the soft solids from pulp and wastewater of olives without the fragments of pits as by-products.

**Fig. 1a:**
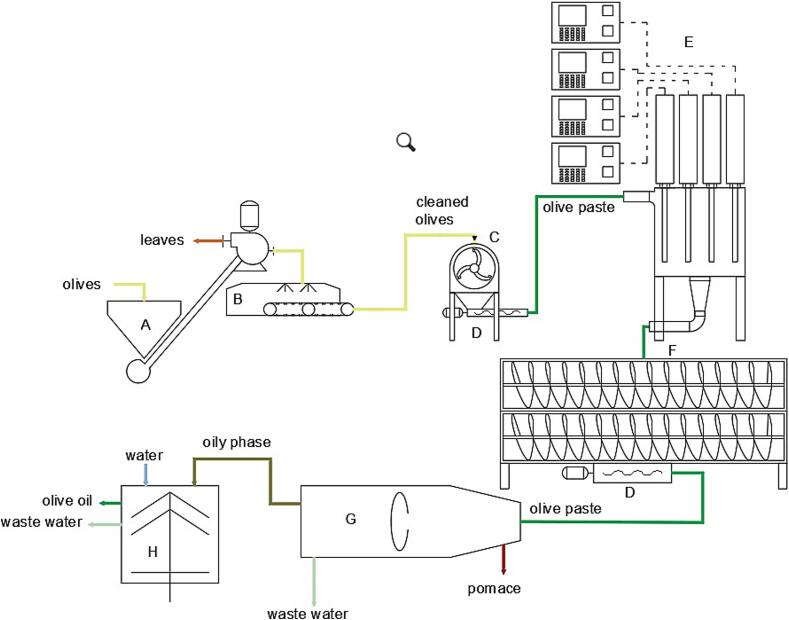
Agricola Malerba − Scheme of olive oil extraction line: A. Defoliator; B. washing machine; C. crusher; D. cavity pump; E. US machine; F. vertical-malaxer; G. horizontal centrifuge; H. vertical centrifuges.

The Frantoio Cutrera mill, Fig. 1b, was composed of a defoliator (mod. Imperatrice; Mercuri, Rosarno, Italy), a washing machine (mod. Poseidon; Mercuri, Rosarno, Italy), a knife crusher (mod. Monogriglia HP; Pieralisi, Jesi, Italy), a group of six malaxer machines each with a capacity of 700 kg, arranged in parallel way (mod. Myblend SX, Pieralisi, Jesi, Italy), a decanter (mod. Leopard 10; Pieralisi, Jesi, Italy) and two vertical plate centrifuges (mod. Marte; Pieralisi, Jesi, Italy). The decanter was set for a 2.5-phases without water added.

**Fig. 1b:**
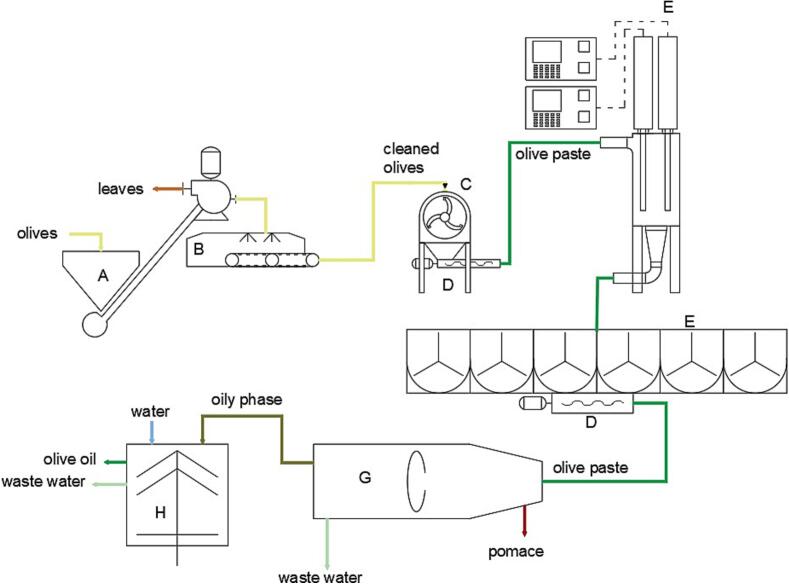
Frantoio Cutrera − Scheme of olive oil extraction line: A. Defoliator; B. washing machine; C. crusher; D. cavity pump; E. US machine; F. 6-malaxer section; G. horizontal centrifuge; H. vertical centrifuges.

In both plants the two wastewaters exiting the decanter (pomace and patè) were combined into a single wastewater to manage the wet pomace.

The extraction plants were both modified with the addition of a US machine, appropriately according to the mill flow rates, for the treatment of the olive paste after the crusher and before the malaxers.

The cultivars used for the tests are different in the two plants: in mill Agricola Malerba had been processed Coratina cultivar with maturity index 2.2, instead in mill Frantoio Cutrera had been processed Nocellara del Belice cultivars with maturity index 2.0.

### 2.2 High power ultrasound (HPU) machine

The US machines installed on existing olive oil mill plants to carry out experimental tests on different olive cultivars were produced by Hielscher Gmbh (Teltow, Germany) and installed by Seneco Scienza (Seneco s.r.l., Milan, Italy).

The US system installed in the Frantoio Cutrera mill consists of 2 UIP4000hdT units, powered by 4  kW each (total 8 kW), two ultrasound generators (mod. RS4d64L4 Cascatrode™, Hielscher Gmbh) working at 20 kHz, and two transducers. Each ultrasound generator was installed in a treatment cell (mod. FC95L5-95F64 Hielscher Gmbh) with a net usable capacity of olive pasta processed of 10.6 kg.

The system installed in the Agricola Malerba mill consists of 4 UIP4000hdT units, powered by 4 kW each (total 16 kW), four ultrasound generators (mod. RS4d64L4 Cascatrode™, Hielscher Gmbh) working at 20  kHz, and four transducers. All four ultrasound generators were installed in a common treatment cell (mod. MSR4 Hielscher Gmbh) with a net usable capacity of olive paste processed of 48.8 kg.

All operative parameters were controlled by a PLC equipped with a touchscreen PLC equipped with a touchscreen, allowing the amplitude to be set between 0 and 100 %. The Cascatrode™ was placed inside a vertical stainless-steel tube (cell).

Both US implants have components made of titanium, ensuring a long lifespan and minimal maintenance costs.

**Fig. 2:**
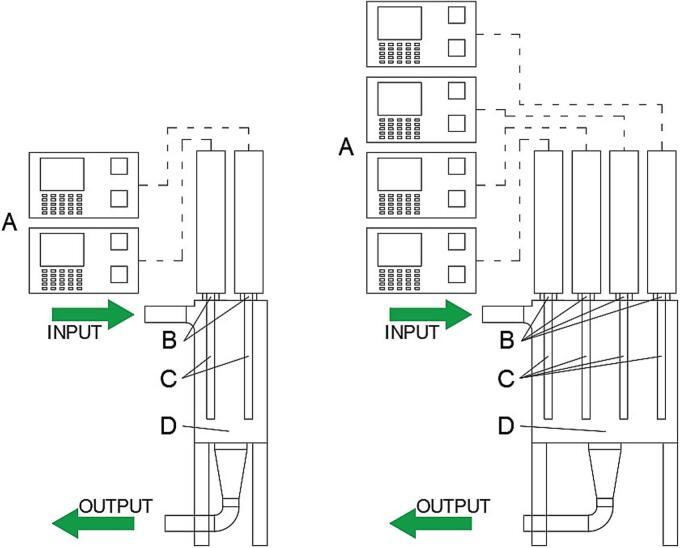
Ultrasound machine: A. generator; B. booster; C. sonotrode; D. flowcell.

The US machine operate in continuous-flow during the upload phase on malaxers. For both plants, the machine was connected to the malaxer through DN90 connections and positioned just before the olive paste entered the malaxer after the crusher. Olive paste flowed into the cell coming from the top side and exited at the bottom side (Fig. 2). A pneumatic valve was installed on the outlet of the US machine to set the pressure in the cell. A pressure probe was also installed on the output section of the US cell, to monitor the olive paste pressure every second. Data were recorded on a SD card installed in the PLC. The pressure value determined the US effect on olive paste, in terms of energy transferred. In fact, the higher the pressure value, the more electric power was adsorbed by the machine, because the US frequency must be constant and to do this, the generator had to spend more energy in case the olive paste required more mechanical resistance due to the higher pressure inside the cell. All parameters (electric power, pressure, amplitude and pulsation frequency) were visible in real-time on the display of the PLC. Considering the chosen frequency and amplitude parameters, the system was able to automatically modulate the electric power used as a function of the pressure detected in the US-cell.

### 2.3 Experimental plant design

Experimental tests were performed in two similar olive oil mills. At the Agricola Malerba mill, olives of the Coratina cultivar mechanically harvested in the municipality of Terlizzi (BA) were processed, while at the Frantoio Cutrera mill, olives of the Nocellara del Belice cultivar mechanically harvested in the municipality of Piano Dell’Acqua (RG) were processed.

For both mills, the olives had been processed within 6 h from the harvest to carry out the tests. In Frantoi Cutrera mill, each configuration was performed five times, processing homogeneous olive batch of 700 kg. In Agricola Malerba mill, each configuration was performed five time by processing homogeneous olive batch of 2800 kg.

Operative parameters concerning the malaxation phase were monitored and recorded during the tests performed for both mills. The olive paste was processed in the malaxer for 25 min at 27 °C.

The US machine, set at a frequency of 20 kHz for the experimental tests, was used in two configurations: ON and OFF. In the ON configuration, 100 % of the power was used to activates the US treatment on the olive paste; in the OFF configuration, the system works in conventional conditions. Considering the net usable capacities of olive paste processed of 48.8 kg and 10.6 kg and the feed flow rate of the ultrasonic cell equal to 5000 kg h^−1^ and 2300 kg h^−1^ for Agricola Malerba mill and Frantoio Cutrera mill, respectively, the ultrasound treatment time of the olive paste was calculated to be 36.8 s and 17.4 s for Agricola Malerba mill and Frantoio Cutrera mill, respectively. The specific energy transferred per kilogram of olive paste was 11.52 kJ kg^−1^ and 12.52 kJ kg^−1^ for Agricola Malerba mill and Frantoio Cutrera mill, respectively.

The decanter mass flow rate was 6500 kg h^−1^ and 2300 kg h^−1^ for Agricola Malerba mill and Frantoio Cutrera mill.

For each test, representative samples were taken of olives in the feed, two-phase pomace exiting the decanter and oil exiting the vertical separator.

### 2.4 Characterization of olive paste

#### 2.4.1 Moisture and oil content in olives and pomace

Moisture content (% w/w) was determined by drying the milled olives or pomace samples taken from the decanter at 105 °C to constant weight. The total oil content was measured following the analytical technique described by Cherubini et al. 2009.

The performance of the two systems was evaluated using the olive oil extractability parameters and the oil content in the pomace and wastewater. Olive oil extractability (E) is defined as the ratio between the percentage of oil extracted from the olives (Poe) by the plant and the percentage of oil contained in the olives (Poo), calculated with the equation:

(1) $$E=\frac{\mathrm{Poe}}{\mathrm{Poo}}*100$$

The oil content in the olives, pomace and wastewater was evaluated according to [17,18].

### 2.5 Olive oil analysis

#### 2.5.1 Solvents and reference compounds

All chemical compounds and solvents used for VOO analyses, such as hydroxytyrosol (3,4-DHPEA), tyrosol (p-HPEA), methanol (HPLC grade), water, isobutyl acetate, chloroform, ethanol, glacial acetic acid, diethyl ether and isooctane (spectrophotometric grade), were supplied by Merck (Merck KGaA, Darmstadt, Germany). Hydrophilic phenols such as oleuropein and ligstroside derivatives (3,4-DHPEA-EDA, 3,4-DHPEA-EA, *p*-HPEA-EDA and *p*-HPEA-EA) and lignans ((+)-pinoresinol and (+)-1-acetoxypinoresinol) were extracted and detected following the method described by [35].

#### 2.5.2 Legal quality parameters

The quality characteristics of olive oil used for the identification of oil category were determined following the Commission Delegated Regulation EU 2022/2104[36]. The analysis was carried out evaluating: acidity (%), peroxide value (mEq O_2_/kg), K_232_, K_268_ or K_270_, and ΔΚ.

#### 2.5.3 Phenolic compounds

An Agilent Technologies system Model 1100 (Agilent Technologies, Santa Clara, CA, USA) equipped with diode array detector (DAD) and fluorescence detector (FLD) was used to perform the HPLC analyses of VOOs. The qualitative and quantitative data of hydrophilic phenols content were evaluated by using a Spherisorb ODS-1 C18 column (Waters, Milford, MA, USA) with the following technical characteristics: 250 mm × 4.6 mm with a particle size of 5 μm[37]. The different oleuropein derivatives, ligstroside derivatives and lignans, extracted with the method described by [38], were expressed in mg kg^−1^ of olive oil.

#### 2.5.4 Volatile compounds

The main compounds responsible of VOO flavor such as C_5_ and C_6_ aldehydes, alcohols and esters were determined with gas chromatographic analysis by using HS-SPME/GC–MS technology equipped with a SPME fiber 2 cm long and 50/30 μm DVB/Carboxen/PDMS (Stableflex; Supelco, Inc., Bellefonte, PA, USA). The qualitative and quantitative data of the different volatile molecules were obtained with Agilent Technologies GC 7890B with “Multimode Injector” (MMI) 7693A (Agilent Technologies, Santa Clara, CA, USA) as reported by Taticchi et al. [38] and expressed as μg kg^−1^ of olive oil.

### 2.6 Statistical analysis

The statistical method used to elaborate the results of chemical analyses of VOO was carried out with the software SigmaPlot V.12.3 (Systat Software Inc., San Jose, CA, USA). ANOVA (Tukey’s test, p < 0.05) was used to determine the statistical differences among the different trials.

## 3 Results and discussion

### 3.1 Impact of US treatment on extractability

Table1 shows the quantitative results obtained in the by-products of the olive oil extraction process. The pomace samples treated with US technology contained significantly lower content of residual olive oil than untreated samples. This demonstrates the ability of US treatment to improve the extraction of olive oil from the matrix, with a significant percentage increase of more than 3 %.

**Table 1:** Quantitative results and process parameters.

Test conditions	Cultivar	POMACE		Extractability
		**Moisture** **(%)**	**Oil** **(%. db)**	**(%)**
CONTROL	Coratina	52.9 ± 1.3 a	4.8 ± 0.4 a	89.0 ± 0.8b
US	Coratina	53.5 ± 0.4 a	3.0 ± 0.3b	92.1 ± 0.7 a
CONTROL	Nocellara del Belice	59.3 ± 1.2 a	4.6 ± 0.1 a	80.4 ± 0.5b
US	Nocellara del Belice	58.7 ± 2.1b	4.0 ± 0.2b	83.2 ± 0.7 a

Different letters in columns denote significant statistical differences (p < 0.05).

US can increase mass transfer during extraction of plant materials to improve extraction and shorten extraction time[39].

### 3.2 Effects of the ultrasound treatment and the HPU machine installation position on the olive oil quality

Both the cultivars, analyzed at two different industrial extraction plants in Puglia (Coratina cv) and Sicily (Nocellara del Belice cv) region, belonged to olive batches of high quality as shown by the data on quality characteristics of the olive oil (Table 2a, Table 2b and Table 2c). The results showed very low levels of free acidity (< 0.3 %) and peroxide values (< 6.3 meq O_2_ kg^−1^) in all the oils analyzed. Even the spectrophotometric analysis of K_232_ and K_270,_ were abundantly lower than the limits of EVOO category showing, both for control and ultrasound samples, high quality of the final product. The data were not significatively influenced by the ultrasound treatment both for the two cultivars Coratina and Nocellara. Even if the use of ultrasounds in aqueous matrix can be able to induce free radical formation in consequence of thermal dissociation of water vapor into hydroxyl radicals (•OH) and hydrogen atoms (•H) in response the acoustic cavitation, the parameters of EVOO oxidative stability were unchanged after US treatment probably due to the presence of high concentration of hydrophilic phenols characterized by an intense free radical scavenging activity (Akti and Yildiz, 2025; [40].

**Table 2a:** Standard virgin olive oil parameters specified by the IOC (2017) – Coratina (20–12-2021).

Test conditions	Free acidity (%)	Peroxide value (meq O_2_ kg^−1^)	K232	K270	ΔK
Legal limits for EVOO	< 0.8	≤ 20	≤ 2.50	≤ 0.22	≤ 0.01
Control	0.25 ± 0.03 *a*	6.1 ± 0.8 a	1.64 ± 0.07 a	0.13 ± 0.002 a	−0.0003 ± 0.0003 a
US	0.24 ± 0.02 *a*	6.2 ± 0.4 a	1.59 ± 0.03 a	0.13 ± 0.002 a	−0.0003 ± 0.02 a

Different letters in column denotes significative statistical differences among means (p < 0.05).

**Table 2b:** Standard virgin olive oil parameters specified by the IOC (2017) – Coratina (23–12-2021).

Test conditions	Free acidity (%)	Peroxide value (meq O_2_ kg^−1^)	K232	K270	ΔK
Legal limits for EVOO	< 0.8	≤ 20	≤ 2.50	≤ 0.22	≤ 0.01
Control	0.27 ± 0.02 *a*	5.0 ± 0.4 a	1.58 ± 0.07 a	0.14 ± 0.01 a	−0.0004 ± 0.0002 a
US	0.25 ± 0.01 *a*	5.7 ± 0.4 a	1.55 ± 0.04 a	0.14 ± 0.02 a	−0.0004 ± 0.0002 a

Different letters in column denotes significative statistical differences among means (p < 0.05).

**Table 2c:** Standard virgin olive oil parameters specified by the IOC (2017) – Nocellara del Belice.

Test conditions	Free acidity (%)	Peroxide value (meq O_2_ kg^−1^)	K232	K270	ΔK
Legal limits for EVOO	< 0.8	≤ 20	≤ 2.50	≤ 0.22	≤ 0.01
Control	0.20 ± 0.01 *a*	8.0 ± 0.7 a	1.56 ± 0.06 a	0.11 ± 0.01 a	−0.002 ± 0.0004 a
US	0.19 ± 0.01 *a*	7.1 ± 0.9 a	1.57 ± 0.02 a	0.11 ± 0.02 a	−0.003 ± 0.001 a

Different letters in column denotes significative statistical differences among means (p < 0.05).

As reported by other authors the ultrasound extraction activity could have a significant effect on the phenolic composition of EVOO, increasing the extractability of intracellular matrix and improving the solubilization process of bioactive compounds into the oily phase[25,[20], [21], [22]]. Although others studies showed a lower effect or no effect on the phenolic fraction, as consequence of the influence of olive fruits of different genetic origin and/or due to the effect of olive ripening that could strongly reduce the degradation impact of the cavitation phenomenon on olive tissues softened by the activity of endogenous enzymes at advanced state of maturity[41,42]. The two different batches of Coratina cv showed (Table 3a and Table 3b) a significant increase of total phenols when ultrasound was used for olive oil extraction, with an enhancement of 16.1 % and 19.7 % respectively for the first and second olive batch. The improvement of phenolic composition was mainly due to the increase of oleacein content with the first olive batch that also showed a higher value of oleuropein aglycon (46 %). The ligstroside derivatives such as olecoanthal, ligstroside aglycon and tyrosol seem not to be influenced by the US treatment, with the only exception of tyrosol of the second batch that showed a significant reduction. (+)-1-Acetoxypinoresinol and (+)-pinoresinol, the two lingans present in the EVOOs also showed a high stability when compared to the control test and are not influenced by the technological extraction process as reported in literature[[20], [21], [22],^42^]. On the contrary, the phenolic composition of Nocellara EVOOs did not show (Table 3c) any significant changes when the new technology was used with a slight increase of oleuropein derivatives that was not statistically significant. The absences of improving effect on the phenolic fraction could be due to an advanced stage of olive ripening characterized by an intense activity of enzymes such as pectinesterase, polygalacturonase and methylesterase, which are able to release high concentrations of polyphenols in the liquid matrix of olive paste during the extraction process, annulling the effects of acoustic cavitation[43,44,45].

**Table 3a:** Phenolic composition of EVOOs. Data expressed as mg kg^−1^ – Coratina (20–12-2021).

	Control			US		
3,4-DHPEAa	4.3	±	1.2 a	4.2	±	0.4 a
*p-*HPEA	3.5	±	0.5 a	3.7	±	0.3 a
Vanilic acid	0.2	±	0.04 a	0.1	±	0.01 a
3,4-DHPEA-EDA	221.1	±	5.0b	247.0	±	14.6 a
*p*-HPEA-EDA	100.2	±	6.5 a	104.8	±	9.9 a
(+)-1-acetoxypinoresinol	6.8	±	0.6 a	7.2	±	0.4 a
(+)-pinoresinol	13.2	±	1.2 a	12.6	±	1.1 a
3,4-DHPEA-EA	143.1	±	16.7b	208.9	±	13.7 a
Ligstroside aglycone	32.3	±	6.2 a	20.8	±	6.1 a
**Total phenols**	524.7	±	25.8b	609.2	±	26.9 a
Oleuropein derivatives	368.5	±	17.4b	460.1	±	20.0 a
Ligustrside derivatives	136.0	±	9.0 a	129.3	±	11.6 a
Lignans	20.1	±	1.3 a	19.7	±	1.2 a

Different letters in rows denote significant statistical differences (p<0.05).

**Table 3b:** Phenolic composition of EVOOs. Data expressed as mg kg^−1^ – Coratina (23–12-2021).

	Control		US			
3,4-DHPEAa	5.2	±	0.01 a	4.7	±	0.6 a
*p-*HPEA	14.0	±	0.02 a	2.9	±	0.2b
Vanilic acid	0.2	±	0.003 a	0.1	±	0.02 a
3,4-DHPEA-EDA	118.9	±	7.0b	230.2	±	6.0 a
*p*-HPEA-EDA	140.1	±	8.3 a	133.4	±	11.6 a
(+)-1-acetoxypinoresinol	25.1	±	0.9 a	23.7	±	3.6 a
(+)-pinoresinol	10.0	±	0.2 a	12.8	±	1.2 a
3,4-DHPEA-EA	162.5	±	3.4 a	172.7	±	6.0 a
Ligstroside aglycone	26.3	±	0.3 a	20.9	±	4.5 a
**Total phenols**	502.3	±	11.9b	601.3	±	16.9 a
Oleuropein derivatives	286.7	±	7.8b	407.6	±	8.4 a
Ligustrside derivatives	180.4	±	8.3 a	157.1	±	12.4 a
Lignans	35.1	±	0.9 a	36.5	±	3.8 a

Different letters in rows denote significant statistical differences (p<0.05).

**Table 3c:** Phenolic composition of EVOOs. Data expressed as mg kg^−1^ – Nocellara del Belice.

	Control			US		
3,4-DHPEAa	2.2	±	0.3 a	1.9	±	0.4 a
*p-*HPEA	1.6	±	0.4° a	1.5	±	0.4 a
Vanilic acid	0.7	±	0.02 a	0.6	±	0.2 a
3,4-DHPEA-EDA	250.1	±	18.0 a	261.5	±	16.8 a
*p*-HPEA-EDA	46.0	±	2.7 a	47.5	±	1.4 a
(+)-1-acetoxypinoresinol	28.0	±	0.4 a	28.0	±	1.0 a
(+)-pinoresinol	8.8	±	0.2 a	8.2	±	0.2 a
3,4-DHPEA-EA	57.1	±	1.5 a	62.4	±	5.2 a
Ligstroside aglycone	6.5	±	0.2 a	6.4	±	0.2 a
Luteolin	0.4	±	0.01 a	0.4	±	0.01 a
**Total phenols**	401.3	±	18.4 a	418.2	±	18.4 a
Oleuropein derivatives	309.4	±	18.1 a	325.7	±	17.6 a
Ligustrside derivatives	54.0	±	2.8 a	55.4	±	1.4 a
Lignans	36.8	±	0.5 a	36.2	±	0.1 a

Different letters in rows denote significant statistical differences (p < 0.05).

As reported by several authors, the non-thermal ultrasound treatment do not influence the activity of lipoxygenase pathway and the development of volatile compounds during the EVOO extraction process. Even this study, carried out with a four-probe industrial ultrasound plant, confirmed previous scientific researches. Table 4a, Table 4b, Table 4c did not show significant modifications of volatile profile of the final product extracted with both Coratina or Nocellara cultivars. From a qualitative point of view, can be reported only some significant exceptions such as: a reduction of hexanal for the second Coratina ripening stage and a decrease of (*Z*)-3-Hexen-1-ol for both batches of the same cultivar when compared with the control test. On the contrary, Nocellara showed a high quantitative and qualitative stability for all the volatile molecules belonged to C_5_ and C_6_ saturated and unsaturated aldehydes, alcohols and esters responsible of EVOO sensory notes.

**Table 4a:** Volatile compounds detected in olive oils – Coratina (20–12-2021).

	Control		US					
**Aldehydes**								
Pentanal	50	±	8	*a*	62	±	13	*a*
(*E*)-2-Pentenal	12	±	5	*a*	12	±	5	*a*
Hexanal	502	±	22	*a*	496	±	43	*a*
(*E*)-2-Hexenal	17,929	±	1072	*a*	17,321	±	1296	*a*
(*E.E*)-2.4-Hexadienal	48	±	9	*a*	56	±	19	*a*
**Σ of the aldehydes at C_5_ and at C_6_**	18,541	±	1077	*a*	17,947	±	1296	*a*
**Alcohols**								
1-Pentanol	72	±	4	*a*	56	±	6	*b*
1-Penten-3-ol	168	±	15	*a*	146	±	11	*a*
(*E*)-2-Penten-1-ol	11	±	1	*a*	12	±	2	*a*
(*Z*)-2-Penten-1-ol	147	±	10	*a*	136	±	9	*a*
1-Hexanol	6009	±	564	*a*	5898	±	186	*a*
(*E*)-2-Hexen-1-ol	6707	±	339	*a*	6680	±	277	*a*
(*Z*)-3-Hexen-1-ol	1571	±	127	*a*	1300	±	107	*b*
**Σ of alcohols at C_5_ and at C_6_**	14,684	±	671	*a*	14,228	±	350	*a*
**Esters**								
Hexyl acetate	59	±	3	*a*	55	±	5	*a*
(*Z*)-3-Hexenyl acetate	139	±	14	*a*	135	±	12	*a*
**Σ of esters at C_6_**	198	±	14	*a*	190	±	13	*a*
**Ketones**								
3-Pentanone	476	±	15	*a*	463	±	37	*a*
1-Penten-3-one	4	±	1	*a*	4	±	1	*a*
6-Methyl-5-hepten-2-one	19	±	2	*a*	14	±	1	*b*
**Σ sof ketones at C_5_ and at C_8_**	499	±	15	*a*	481	±	37	*a*

Different letters in rows denotes significant statistical differences at p<0.05 (Tuckey’s test).

**Table 4b:** Volatile compounds detected in olive oils – Coratina (20–12-2021).

	Control				US			
**Aldehydes**								
Pentanal	83	±	2	*a*	77	±	15	*a*
(*E*)-2-Pentenal	14	±	2	*a*	24	±	1	*a*
Hexanal	1733	±	91	*a*	1101	±	75	*b*
(*E*)-2-Hexenal	15,133	±	261	*a*	15,109	±	377	*a*
(*E.E*)-2.4-Hexadienal	68	±	5	*a*	76	±	7	*a*
**Σ of the aldehydes at C_5_ and at C_6_**	17,030	±	276	*a*	16,386	±	385	*a*
**Alcohols**								
1-Pentanol	62	±	13	*a*	57	±	5	*b*
1-Penten-3-ol	21	±	6	*a*	18	±	2	*a*
(*E*)-2-Penten-1-ol	145	±	24	*a*	169	±	11	*a*
(*Z*)-2-Penten-1-ol	150	±	13	*a*	183	±	11	*a*
1-Hexanol	6084	±	569	*a*	5706	±	55	*a*
(*E*)-2-Hexen-1-ol	4761	±	157	*a*	4424	±	173	*a*
(*Z*)-3-Hexen-1-ol	1862	±	210	*a*	1442	±	81	*b*
**Σ of alcohols at C_5_ and at C_6_**	13,084	±	627	*a*	12,000	±	200	*a*
**Esters**								
Hexyl acetate	154	±	12	*a*	140	±	3	*a*
(*Z*)-3-Hexenyl acetate	630	±	39	*a*	548	±	51	*a*
**Σ of esters at C_6_**	784	±	41	*a*	687	±	51	*a*
**Ketones**								
3-Pentanone	161	±	16	*a*	100	±	8	*b*
1-Penten-3-one	49	±	7	*a*	34	±	4	*a*
6-Methyl-5-hepten-2-one	24	±	2	*a*	22	±	4	*a*
**Σ sof ketones at C_5_ and at C_8_**	233	±	17	*a*	155	±	10	*b*

Different letters in rows denotes significant statistical differences at p < 0.05 (Tuckey’s test).

**Table 4c:** Volatile compounds detected in olive oils – Nocellara del Belice.

	Control				US			
**Aldehydes**								
Pentanal	107	±	3	*a*	115	±	9	*a*
(*E*)-2-Pentenal	26	±	4	*a*	27	±	3	*a*
Hexanal	1024	±	19	*a*	1052	±	43	*a*
(*E*)-2-Hexenal	3750	±	320	*a*	3793	±	299	*a*
(*E.E*)-2.4-Hexadienal	82	±	13	*a*	76	±	5	*a*
**Σ of the aldehydes at C_5_ and at C_6_**	4989	±	321	*a*	5062	±	302	*a*
**Alcohols**								
1-Pentanol	147	±	18	*a*	156	±	21	*a*
1-Penten-3-ol	549	±	33	*a*	548	±	32	*a*
(*E*)-2-Penten-1-ol	39	±	6	*a*	47	±	5	*a*
(*Z*)-2-Penten-1-ol	327	±	30	*a*	363	±	18	*a*
1-Hexanol	1040	±	32	*a*	1090	±	25	*a*
(*E*)-2-Hexen-1-ol	1081	±	20	*a*	1022	±	26	*a*
(*Z*)-3-Hexen-1-ol	1340	±	41	*a*	1290	±	15	*a*
**Σ of alcohols at C_5_ and at C_6_**	4522	±	74	*a*	4516	±	58	*a*
**Esters**								
Hexyl acetate	20	±	3	*a*	20	±	1	*a*
(*Z*)-3-Hexenyl acetate	76	±	7	*a*	77	±	6	*a*
**Σ of esters at C_6_**	95	±	8	*a*	98	±	6	*a*
**Ketones**								
3-Pentanone	771	±	30	*a*	752	±	14	*a*
1-Penten-3-one	58	±	4	*a*	60	±	5	*a*
6-Methyl-5-hepten-2-one	10	±	1	*a*	10	±	2	*a*
**Σ sof ketones at C_5_ and at C_8_**	839	±	30	*a*	822	±	15	*a*

Different letters in rows denotes significant statistical differences at p < 0.05 (Tuckey’s test).

## 4 Conclusions

The ultrasounds system proved to be easy to install in existing olive oil mills, without modifying the layout of the plant, and considering the small size and the easy coupling with flanges on the olive paste transfer pipe between the crusher and the malaxers.

The analyses, concerning the first-time use of an ultrasound system at industrial scale, showed a significant impact on the phenolic composition of Coratina EVOOs, improving the health properties of the final product with an increase of about 100 mg kg-1. The effect of cavitation induced by ultrasound technology was highly reduced when the extraction process was carried out processing an early cultivar, Nocellara del Belice, at a later ripening stage, when the olive tissues were already softened by maturation enzymes of the olive fruit. The new technology did not alter the legal quality parameters and the main volatile compounds responsible of EVOOs flavour.

## CRediT authorship contribution statement

**Antonia Tamborrino:** Data curation, Conceptualization. **Gianluca Veneziani:** Writing – original draft, Methodology. **Alessandro Leone:** Writing – review & editing. **Antonio Berardi:** Writing – original draft, Supervision. **Sonia Esposto:** Validation, Investigation. **Maurizio Servili:** Supervision.

## Declaration of competing interest

The authors declare that they have no known competing financial interests or personal relationships that could have appeared to influence the work reported in this paper.
